# Identification of potential flavonoids strongly correlated with bitterness and astringency in quinoa seedling vegetables via metabolomics and electronic tongue

**DOI:** 10.1016/j.fochx.2026.104442

**Published:** 2026-09-11

**Authors:** Jie Yang, Yuanyuan Zuo, Chen Chen, Hanxue Li, Xuqin Wang, Guofei Jiang, Yuming Xie, Huanyao Xia, Jianlong Zhou, Lurongrong Gao, Yutao Bai, Chuanli Zhang, Peng Qin

**Affiliations:** a College of Agronomy and Biotechnology, Yunnan Agricultural University, Kunming, Yunnan 650200, China; b College of Tropical Crops, Yunnan Agricultural University, Pu'er, Yunnan 665099, China

**Keywords:** Quinoa seedling vegetables, Metabolome, Electronic tongue, Bitter taste, Astringent taste, Flavonoids

## Abstract

Quinoa (*Chenopodium quinoa* Willd.) seedlings are nutrient-dense leafy vegetables, but their inherent bitterness and astringency limit consumer acceptance. The chemical basis underlying these sensory attributes remains largely unclear. In this study, four quinoa seedling varieties differing in seed coat colour were evaluated using electronic tongue analysis in combination with comprehensive metabolomics. Among the detected flavor attributes, bitterness and astringency were the most prominent in the four identified quinoa seedling varieties. Meanwhile, 2060 metabolites were identified across the four varieties, with differential metabolites predominantly enriched in flavonoids. Correlation analysis revealed significant associations between flavonoids and bitter, astringent sensory traits of quinoa seedlings vegetables, indicating a potential connection to unpleasant taste perception. Among these flavonoids, quercetin and kaempferol glycoside derivatives showed the strongest correlation with bitter and astringent sensory attributes. This study deepens our knowledge of flavor-related metabolites and offers correlational references for breeding low-bitterness quinoa varieties.

## Highlights

• Bitter–astringent signals of quinoa seedlings change synchronously across samples.
• Flavonoids in quinoa seedlings may relate to its bitter–astringent taste.
• Quercetin & kaempferol glycosides are key candidates for bitter–astringent flavor.
• This work offers references for breeding low bitter–astringent quinoa seedlings.

## Graphical abstract

**Figure:**
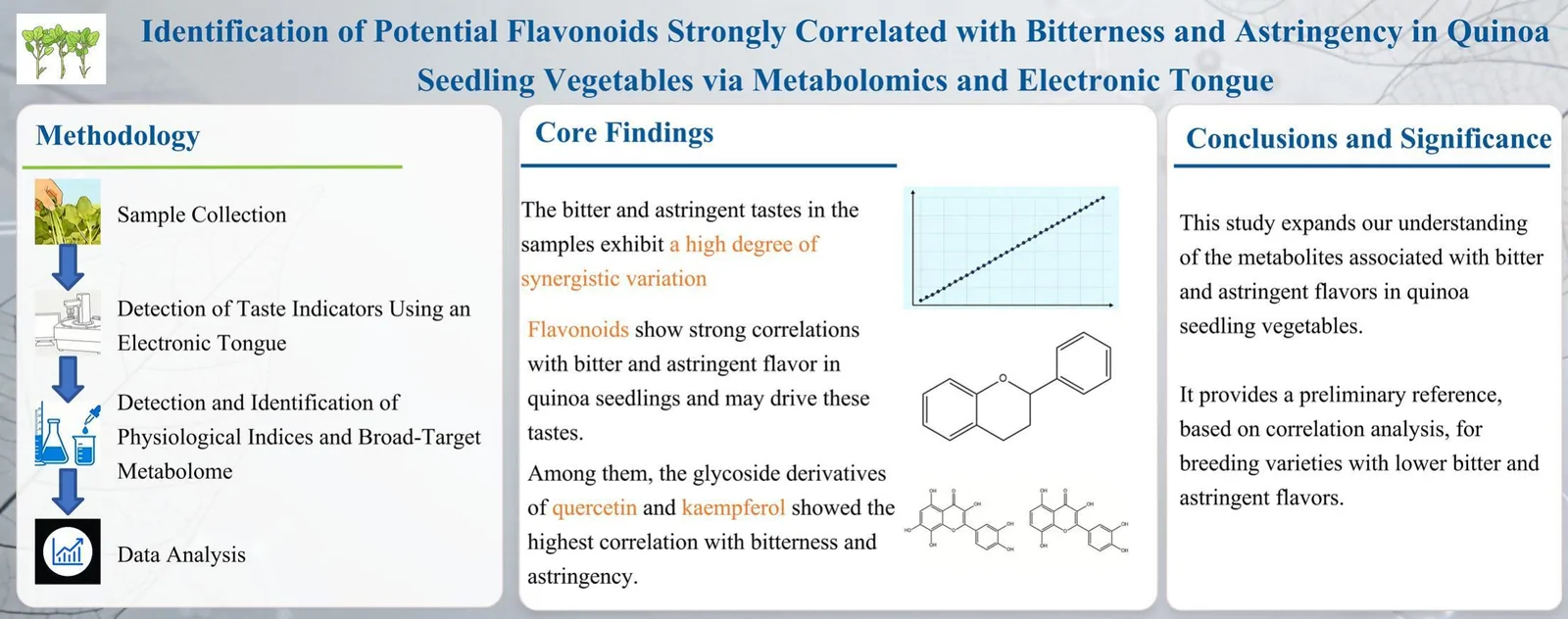

## 1 Introduction

Quinoa (*Chenopodium quinoa* Willd.) is a pseudocereal originating from the Andean region and is widely recognized for its exceptional nutritional value and stress tolerance (Baldera-Chapoñan et al., 2025). Owing to its balanced amino acid profile, gluten-free properties, and rich phytochemical composition, quinoa has been promoted as a functional food crop with global relevance for food security and human health (Vega-Gálvez et al., 2010). The protein content of quinoa seeds ranges from 12 to 23%, which is comparable to that of milk and superior to that of wheat, maize, and rice (Zhang et al., 2026). Quinoa is also a complete protein source, containing all essential amino acids, including lysine, methionine, and threonine (Zhang et al., 2026). It is also rich in lipids, dietary fibers, vitamins, and minerals, which have positive effects on metabolism, the cardiovascular system, and gastrointestinal health (Vilcacundo & Hernández-Ledesma, 2017). Furthermore, quinoa is naturally gluten-free, making it a viable and nutritious alternative for individuals with celiac disease or wheat allergies (Zevallos et al., 2014). Accordingly, quinoa is recognized as an emerging functional food. The United Nations designated 2013 as the International Year of Quinoa (IYQ) to advance human nutrition, enhance food security, and contribute to the achievement of the Millennium Development Goals (MDGs) (Vega-Gálvez et al., 2010).

As an emerging crop, quinoa is currently primarily utilised for its grains. However, the species can also be cultivated as a leafy green vegetable with a comparable high nutritional value (Gómez et al., 2024). Leafy green vegetables refer to a category of vegetables with leaves, petioles, or tender stems as their primary edible organs, and they are distinguished by their high nutrient density (Pathan et al., 2019). Compared with its grains, quinoa seedling vegetables exhibit higher levels of protein, dietary fibre, minerals (e.g., calcium, potassium, magnesium), and total essential amino acids (Pathan et al., 2024). Quinoa seedling vegetables also contain bioactive compounds such as phenolics, flavonoids, and carotenoids, which exhibit health-promoting effects including antioxidant, antibacterial, anticancer, hypoglycemic, and lipid-lowering activities (Pathan & Siddiqui, 2022). Its total dietary fibre content is also significantly higher than that of quinoa grains, which contributes to gut health; its fatty acid profile is dominated by linoleic acid and α-linolenic acid, conferring potential cardiovascular protective effects; and its compatibility with low-calorie diets offers a novel dietary option for individuals with diabetes and those pursuing weight management (Gómez et al., 2024). In conclusion, quinoa seedling vegetables are high-quality leafy vegetables that combine nutritional value and functional properties, thereby supporting healthy dietary patterns. Overall, dedicated research on quinoa seedling vegetables remains limited, and investigations into their sensory properties (taste profiles) are still in the preliminary stage.

However, the sensory attributes (taste) of vegetables are among the core determinants of consumer acceptance and are closely associated with their metabolite composition. For example, free amino acids contribute to the perception of sweetness and umami, while organic acids modulate sourness perception (Chen & Zhang, 2007; Jia et al., 2022). Alkaloids, terpenoids, and flavonoids are associated with bitter taste (Liu et al., 2024), whereas polyphenols are linked to astringency (Liu et al., 2023). Notably, bitter and astringent sensory attributes exert the greatest influence on consumer acceptance of vegetables. These two textural attributes compromise the edibility and consumer acceptability of quinoa products, thereby significantly impeding their large-scale cultivation and industrial applications (Li et al., 2025; Suárez-Estrella et al., 2018). Research on quinoa seedlings as leafy vegetables has demonstrated that low-bitterness varieties can directly serve as a substitute for spinach in the leafy vegetable market, applicable to multiple consumption methods, including raw eating (salads), stir-frying, and juicing (Ding et al., 2025). Consequently, investigations into the bitter and astringent taste profiles of quinoa seedling vegetables are critical to their market promotion.

Advances in analytical technologies now enable comprehensive profiling of taste-related metabolites. Widely targeted metabolomics using UPLC-MS/MS enables high-throughput identification and quantification of secondary metabolites, offering insights into metabolic variation across varieties (Singh et al., 2022). Meanwhile, the electronic tongue can simulate the perception of biological senses and enable objective analysis; compared with traditional detection technologies, it offers higher speed, objectivity, and efficiency (Lu et al., 2022). By combining broad-target metabolomics and electronic tongue technologies and applying a multi-source data fusion strategy, the recognition of food categories and quality prediction can be improved. Currently, this integrated analysis method has been applied to agricultural research (An et al., 2022; Cai et al., 2024).To screen metabolic substances associated with the bitter and astringent flavors of quinoa seedling vegetables, this study combined electronic tongue detection with metabolomics to analyze metabolic differences among quinoa seedling vegetable varieties and to further explore the biochemical basis underlying their bitter and astringent flavor characteristics.

Existing studies have demonstrated that quinoa varieties with differently coloured seeds exhibit significant variation in metabolite composition and content, particularly in amino acids, alkaloids, organic acids, and tannins (Liu et al., 2022; Yang et al., 2024). Accordingly, in this study, four quinoa cultivars with different seed coat colors (white, yellow, red, and black) were selected. Given that their plant height, fresh weight, and dry weight were similar at the seedling stage, we employed a combined approach of widely-targeted metabolomics and electronic tongue technology to systematically compare the metabolite profiles and bitter/astringent intensities of their sprouts, thereby exploring the intrinsic relationship between bitterness/astringency and their corresponding metabolites. The research results can provide a reference and theoretical basis for breeding quinoa seedling vegetables and controlling their taste and quality.

## 2 Materials and methods

### 2.1 Plant materials

In this study, four quinoa varieties independently bred by Yunnan Agricultural University were selected as experimental materials. These varieties differ in seed colour (red, white, yellow, and black) but exhibit comparable plant height, fresh weight, and dry weight at the seedling stage. Uniformly sized and physiologically consistent seeds were selected from the four target strains, and the planting experiment was conducted in the intelligent greenhouse of the Modern Agricultural Education and Research Base, Yunnan Agricultural University (102°41′E, 25°20′N). Seedlings were cultivated under conventional cultivation and management practices, with controlled conditions including an average temperature of 25 °C, an average relative humidity of 60%, a daily sunshine duration of 7 h, and a growing medium composed of loam, humus, and organic fertilizer at a ratio of 1:0.6:0.4. During sowing, seeds were uniformly dibbled into cylindrical pots (20 cm in diameter). Each pot contained 5 sowing holes, spaced 5 cm apart, with 5 seeds sown per hole. Seed emergence time was recorded, and 30 days post-emergence, independent plants were randomly selected from each of the four varieties for sampling. The aboveground parts of the plants were sampled; immediately after sampling, the samples were snap-frozen in liquid nitrogen and stored at −80 °C for subsequent analysis. The four strains were categorised by seed colour into Group R (quinoa seedling vegetables grown from red seeds), Group W (from white seeds), Group Y (from yellow seeds), and Group B (from black seeds). Each group comprised three biological replicates, resulting in a total of 12 samples.

### 2.2 Determination of morphological and physiological traits

The plant height of quinoa seedling vegetables was measured using a ruler, defined as the straight-line distance from the seedling base to the tip of the uppermost fully expanded leaf. For each plant to be tested, fresh weight was directly measured using an electronic balance (Model AL104, Mettler-Toledo Instruments (Shanghai) Co., Ltd., precision: 0.1 mg). Subsequently, the plants were transferred to an electric blast drying oven (Model 101-3AB, Tianjin Tester Instrument Co., Ltd.), blanched at 105 °C for 30 min to inactivate enzymes, and then dried at 70 °C until a constant weight was achieved. After cooling to room temperature, the dry weight was weighed and recorded.

Total alkaloids were quantified using a commercial kit purchased from Beijing Boxbio Science & Technology Co., Ltd. (http://www.boxbio.cn). For flavonoids, total phenols, and saponins, quantification was conducted with kits supplied by Wuhan Pyt Bioengineering Co., Ltd. (Wuhan, China; http://www.pytbio.com). All assays were performed strictly according to the manufacturer's instructions.

### 2.3 Electronic tongue taste measurement

In this study, taste profiling was performed using the INSENT SA402B electronic tongue system. A 1:10 (*w*/*v*) sample-to-ultrapure water ratio was used for extraction: 20 g of quinoa sample was mixed with 200 mL of ultrapure water, then shaken for 30 min. After standing, the mixture was filtered through filter paper, and the resulting supernatant was collected for subsequent electronic tongue analysis. Preprocessed samples were loaded onto the automatic sampler. Specialised taste sensors targeting sweet, sour, umami, salty, and bitter-astringent tastes were employed, and taste signals were captured by the integrated pattern recognition system, transmitted to the data acquisition processor, and subsequently analyzed using the manufacturer-provided software to generate the corresponding taste profile data. Prior to sensor testing, a rigorous cleaning and activation protocol was strictly implemented: First, place the positive and negative sensors, respectively, in the corresponding cleaning solutions for 90 s; then clean them with the two reference solutions for 120 s each in sequence. After the sensors return to their original positions and balance for 30 s, start the formal test. Each test lasts 30 s. After the test, the sensors need to be quickly cleaned in each of the two reference solutions for 3 s, and then immersed in new reference solutions for a 30-s aftertaste test. Given the prolonged on-instrument detection time, to ensure consistent detection status across large batches of samples, samples were processed and subjected to on-instrument detection in batches throughout the experiment.

### 2.4 Broad-targeted metabolomics analysis

Vacuum freeze-drying was employed to lyophilise biological samples in a freeze dryer (Scientz-100F); subsequently, the samples were ground into a fine powder using a ball mill (MM 400, Retsch) at 30 Hz for 1.5 min. An electronic balance (MS105DΜ) was used to weigh 30 mg of sample powder, which was then transferred into 1500 μL of a pre-cooled (−20 °C) 70% methanol-water internal standard extraction solution. For samples weighing less than 30 mg, the volume of the extraction solution was adjusted proportionally to 1500 μL per 30 mg of sample. The mixture was vortexed for 30 s every 30 min, with a total of 6 cycles. Following centrifugation at 12,000 rpm for 3 min, the supernatant was carefully aspirated, filtered through a 0.22 μm pore-size microporous membrane, and the resulting filtrate was collected into an autosampler vial for subsequent ultra-performance liquid chromatography-tandem mass spectrometry (UPLC-MS/MS) analysis.

Sample extracts were analyzed using a UPLC-ESI-MS/MS system (UPLC: ExionLC™ AD, https://sciex.com.cn/) and a tandem mass spectrometry system (https://sciex.com.cn/). Chromatographic separation was performed on an Agilent SB-C18 column (1.8 μm, 2.1 mm × 100 mm). The mobile phase consisted of solvent A (water with 0.1% formic acid) and solvent B (acetonitrile with 0.1% formic acid). Gradient elution was set as: 95% A at first, linearly changed to 5% A within 9 min and held for 1 min, then restored to 95% A within 1.1 min and maintained for 2.9 min. The flow rate was 0.35 mL/min, column temperature 40 °C, and injection volume 2 μL. The effluent was analyzed by an ESI-triple quadrupole-linear ion trap (QTRAP) mass spectrometer.

ESI-MS parameters were optimized as follows: source temperature, 500 °C; ion spray voltage, 5500 V (positive mode) and −4500 V (negative mode); ion source gas I, ion source gas II, and curtain gas were set to 50, 60, and 25 psi, respectively; collision-activated dissociation (CAD) was set to high. Multiple reaction monitoring (MRM) was conducted using a triple quadrupole (QQQ) mass analyzer with medium-pressure nitrogen as collision gas. The declustering potential (DP) and collision energy (CE) for each MRM transition were individually optimized. During each acquisition period, characteristic MRM transitions were monitored based on the elution times of the corresponding metabolites. This method was applied for relative semi-quantification, and full quantitative validation was omitted due to incomplete reference standards for all analytes.

Pooled quality control (QC) samples were prepared by mixing equal volumes of extracts from all quinoa samples. One QC sample was injected every 10 test samples during UPLC-ESI-MS/MS analysis. The tight clustering of QC samples in PCA indicated stable instrument performance and good detection repeatability.

Metabolite identification was performed using a UPLC-ESI-MS/MS system with multiple reaction monitoring (MRM) mode. Metabolites were identified by matching accurate precursor ion *m*/*z*, characteristic fragment ions, retention time and fragmentation patterns against the in-house metabolite database, with cross-verification using public databases including HMDB, METLIN and MassBank. All identified metabolites were further annotated against the KEGG Compound and KEGG Pathway databases.

Broad-target metabolomics data were subjected to unsupervised principal component analysis (PCA) with the prcomp function in R software (www.r-project.org). All data were standardized to unit variance prior to statistical analysis. Hierarchical cluster analysis (HCA) was performed on both samples and metabolites, and the results were visualized as heatmaps with dendrograms. Pearson correlation coefficients (PCCs) among samples were calculated with the cor function in R and presented as heatmaps. All heatmap-related visualizations were accomplished using the R package ComplexHeatmap. In HCA, the unit-variance-standardized metabolite intensities were displayed accordingly. For pairwise group comparisons, differential metabolites were screened according to the following thresholds: variable importance in projection (VIP) >1, absolute log₂ fold change (|log₂FC|) ≥ 1.0, and false discovery rate (FDR)-adjusted *p*-value <0.05. VIP values were obtained from orthogonal partial least squares discriminant analysis (OPLS-DA), which also generated score plots and permutation test plots. OPLS-DA was conducted using the R package MetaboAnalystR. Before modeling, the data were log₂-transformed and mean-centered, and 200 permutation tests were performed to mitigate model overfitting. The characterized metabolites were annotated using the KEGG COMPOUND database (http://www.kegg.jp/kegg/compound/). Annotated metabolites were further mapped to the KEGG PATHWAY database (http://www.kegg.jp/kegg/pathway.html) for metabolic pathway enrichment analysis.

### 2.5 Data analysis methodology

Morphological and physiological data were processed and statistically analyzed using Excel 2021 (Microsoft Corporation, Redmond, WA, USA) and R software (version 4.5.1; R Core Team, 2024). A two-way analysis of variance (ANOVA) was employed to identify significant differences, with statistical significance defined as a *P*-value <0.05. All data presented in the tables are expressed as mean ± standard deviation (SD; *n* ≥ 3).

## 3 Result

### 3.1 Phenotype analysis

**Fig. 1:**
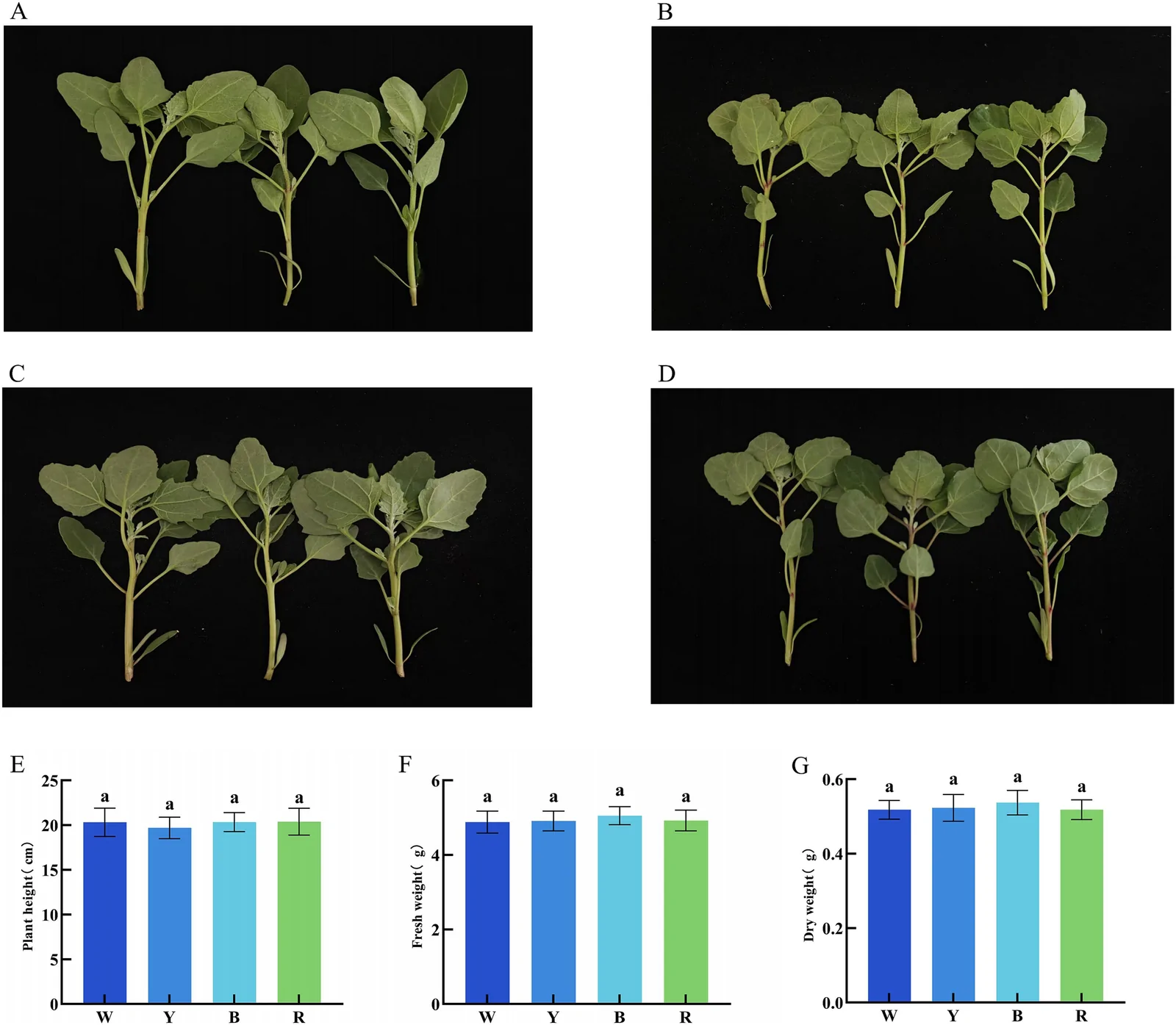
Varietal differences in white, yellow, black, and red quinoa at the seedling stage; A: White-seeded quinoa seedling vegetable (Group W); B: Yellow-seeded quinoa seedling vegetable (Group Y); C: Black-seeded quinoa seedling vegetable (Group B); D: Red-seeded quinoa seedling vegetable (Group R); (E) Plant height comparison of the four quinoa seedling vegetable cultivars; (F) Fresh weight comparison of the four quinoa seedling vegetable cultivars; (G) Dry weight comparison of the four quinoa seedling vegetable cultivars. Different lowercase letters indicate significant differences at the 0.05 level. (For interpretation of the references to colour in this figure legend, the reader is referred to the web version of this article.)

The four quinoa seedling vegetable cultivars employed in the experiment exhibited no significant differences in plant morphology (Fig. 1A–D), nor in plant height, fresh weight, or dry weight (Fig. 1E–G). Individual plant heights ranged from 17 to 23 cm, fresh weights per plant from 4.5 to 5.5 g, and dry weights per plant from 0.45 to 0.57 g. These findings indicate that their core morphological traits during the seedling stage were largely consistent.

### 3.2 Electronic tongue analysis

**Fig. 2:**
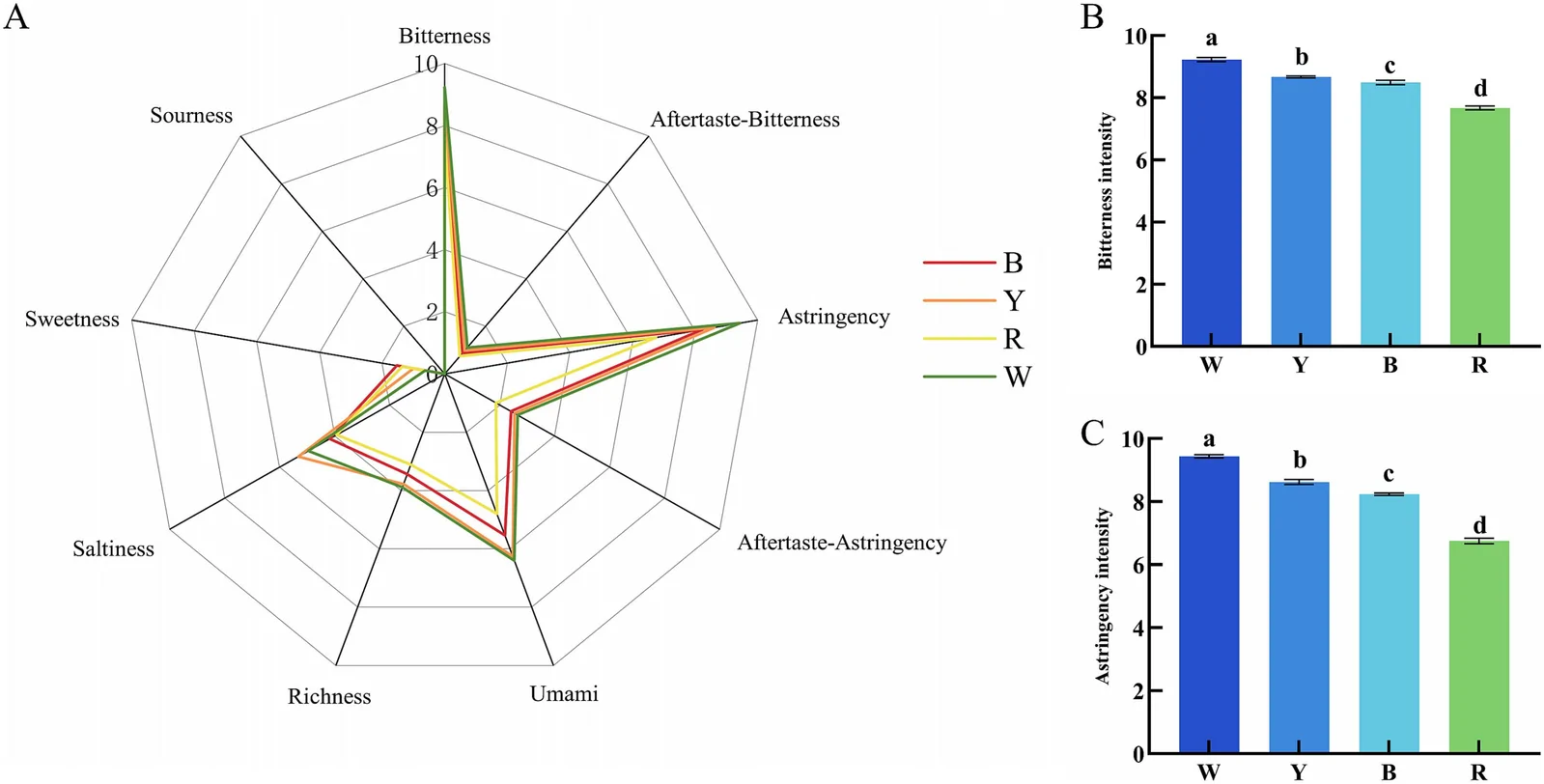
(A) Radar plot illustrating the relative intensities of nine taste attributes; (B) Comparison of bitterness intensity among four quinoa seedling vegetable cultivars; (C) Comparison of astringency among four quinoa seedling vegetable cultivars. Concentric circles of the radar plot represent increasing taste intensity from the inner to the outer layers—data points farther from the center indicate higher perceived intensity of the corresponding taste attribute. Higher values in the bar graphs correspond to stronger taste intensities. Different lowercase letters denote significant differences at the 0.05 level.

Electronic tongue data of the four sample groups were visualized as a radar plot (Fig. 2A). The plot illustrates the presence of nine taste attributes: bitterness, bitter aftertaste, astringency, astringent aftertaste, umami, umami aftertaste, saltiness, sweetness, and sourness. Among these, bitterness and astringency were relatively prominent. Although sweetness was detected, its contribution to the overall taste profile was negligible. To further characterize the differences in bitterness and astringency across the four groups, the corresponding electronic tongue data were presented as bar graphs (Fig. 2B and C). As shown in Fig. 2B, Group W exhibited the highest bitterness intensity, while Group R showed the lowest; pairwise comparisons revealed significant differences in bitterness between all sample groups. Similarly, Fig. 2C shows that Group W had the highest astringency, with Group R again showing the lowest; significant differences in astringency were also observed between all pairs of samples.

### 3.3 Physiological parameter analysis

**Fig. 3:**
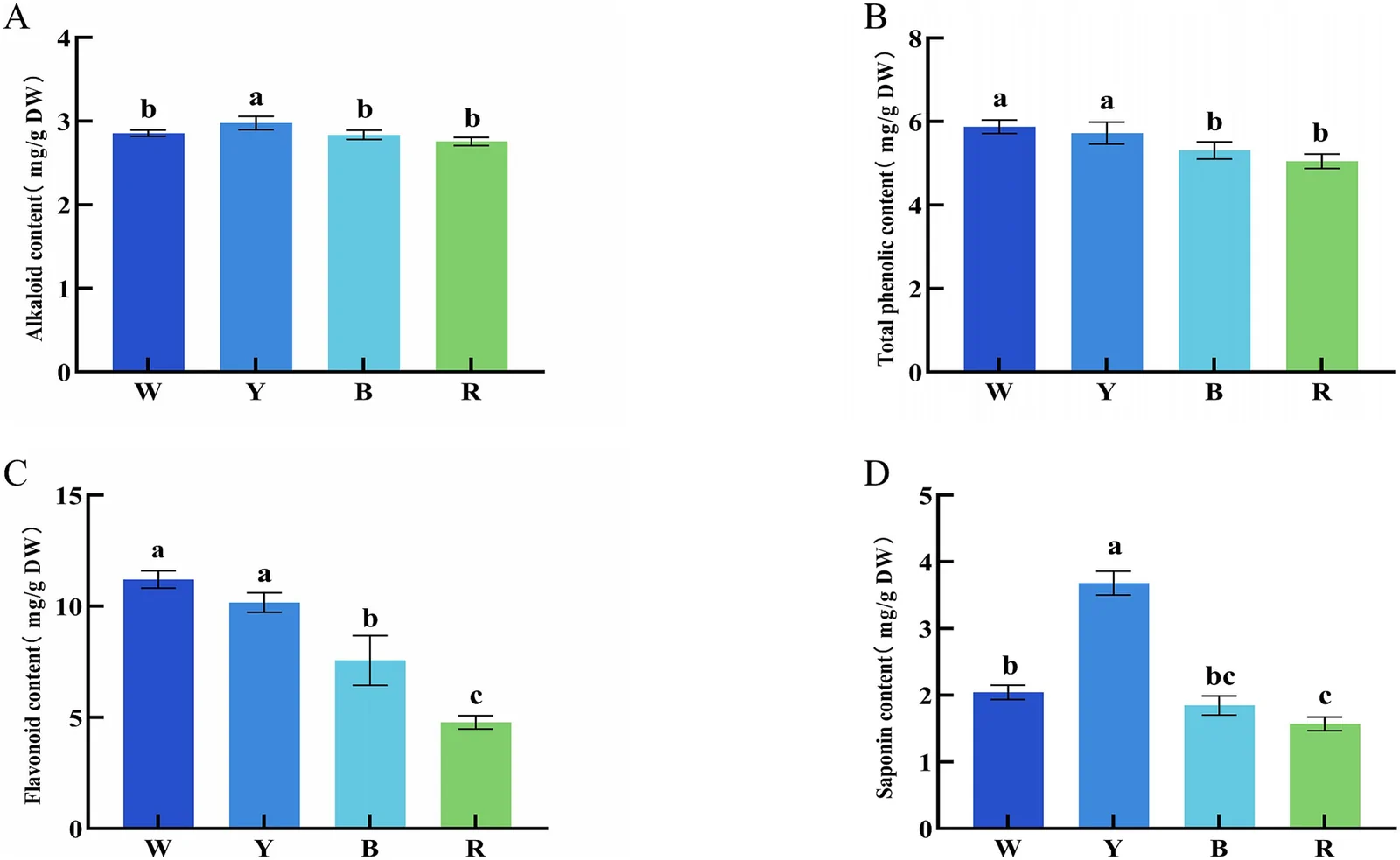
Comparison of Substance Contents Among Groups W, Y, B, and R.(A) Alkaloid content of quinoa seedling vegetables; (B) Total phenolic content of quinoa seedling vegetables; (C) Flavonoid content of quinoa seedling vegetables; (D) Saponin content of quinoa seedling vegetables. Different lowercase letters indicate significant differences at the 0.05 probability level.

The alkaloid content in quinoa seedling vegetables is presented in Fig. 3A. Specifically, the alkaloid content of Group W was 2.86 ± 0.04 mg/g, Group Y was 2.98 ± 0.08 mg/g, Group B was 2.83 ± 0.06 mg/g, and Group R was 2.76 ± 0.05 mg/g. Among these groups, Group Y exhibited the highest alkaloid content, while Group R showed the lowest. No significant differences were observed in alkaloid content between Group W, Group B, and Group R, and all three groups had significantly lower alkaloid content compared to Group Y. The total phenolic content in quinoa seedling vegetables is presented in Fig. 3B. Specifically, the total phenolic content of Group W was 5.89 ± 0.16 mg/g, Group Y was 5.73 ± 0.26 mg/g, Group B was 5.31 ± 0.21 mg/g, and Group R was 5.05 ± 0.17 mg/g. Among these groups, Group W exhibited the highest total phenolic content, while Group R showed the lowest. No significant difference was observed in total phenolic content between Group W and Group Y; similarly, no significant difference was found between Group B and Group R, and both Group B and Group R had significantly lower total phenolic content compared to Group W and Group Y. The flavonoid content in quinoa seedling vegetables is presented in Fig. 3C. Specifically, the flavonoid content of Group W was 11.21 ± 0.40 mg/g, Group Y was 10.17 ± 0.44 mg/g, Group B was 7.56 ± 1.12 mg/g, and Group R was 4.79 ± 0.30 mg/g. Among these groups, Group W exhibited the highest flavonoid content, while Group R showed the lowest. No significant difference was observed in flavonoid content between Group W and Group Y; the flavonoid content of Group B was significantly lower than that of Groups W and Y; and the flavonoid content of Group R was significantly lower than that of the other three groups. The saponin content in quinoa seedling vegetables is presented in Fig. 3D. Specifically, the saponin content of Group W was 2.04 ± 0.11 mg/g, Group Y was 3.68 ± 0.18 mg/g, Group B was 1.84 ± 0.14 mg/g, and Group R was 1.57 ± 0.10 mg/g. Among these groups, Group Y exhibited the highest saponin content, while Group R showed the lowest. The saponin content of Group Y was significantly higher than that of the other groups; the saponin content of Group W was significantly higher than that of Groups B and R, and the saponin content of Group B was significantly higher than that of Group R. Among the four physiological indices, the content trends of total phenols and flavonoids were consistent with the bitterness and astringency trends, which may contribute to the differences in bitterness and astringency among the four varieties.

### 3.4 Metabolomic profiling

**Fig. 4:**
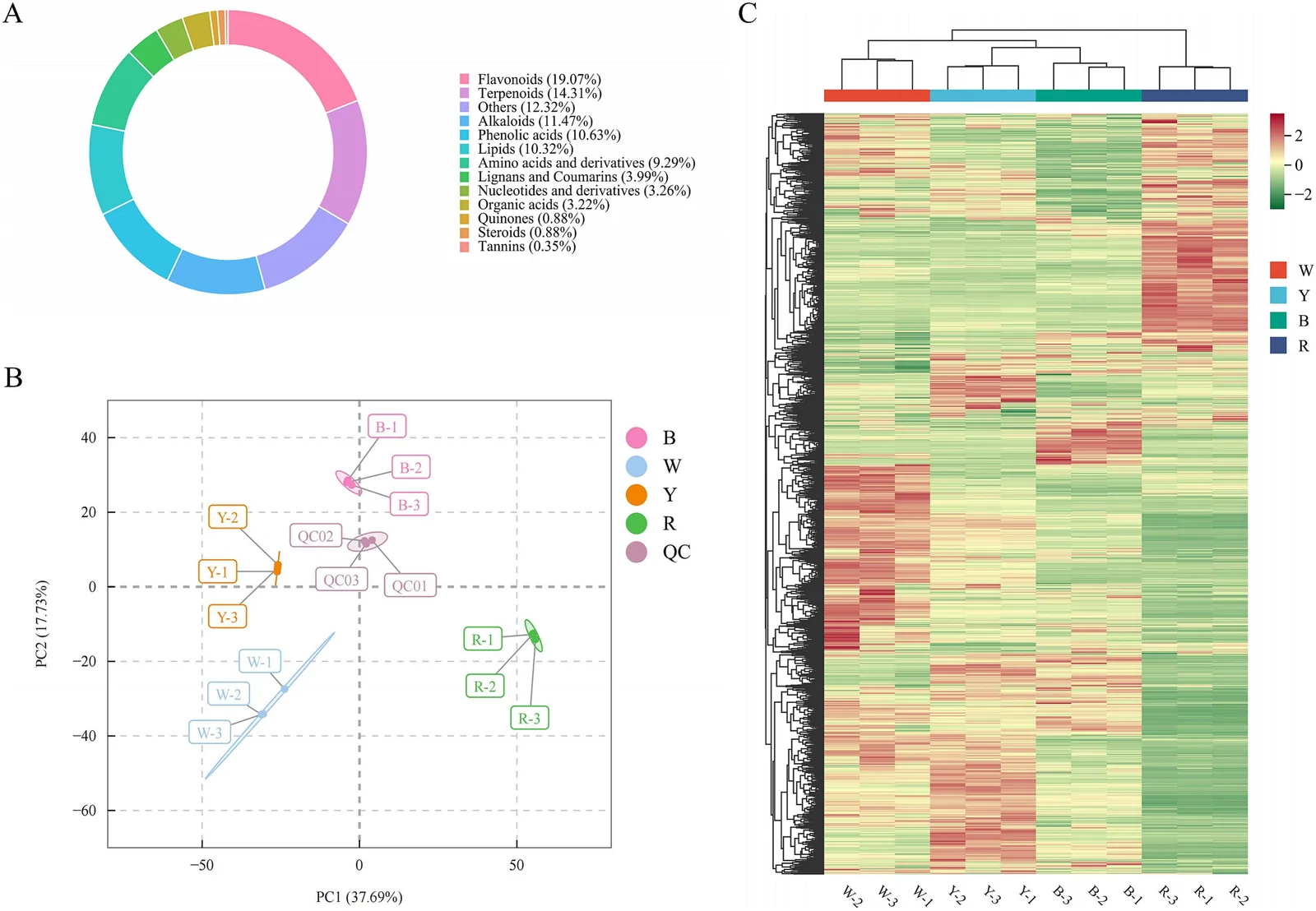
(A) Classification proportion chart of all detected metabolites; (B) Principal component analysis (PCA) score plot for the four sample groups and quality control (QC) samples; (C) Heatmap of differential metabolite expression levels across all samples.

To investigate metabolite differences among quinoa seedling vegetables of different varieties, primary and secondary metabolites were identified in the samples using a widely targeted metabolomics approach. A total of 2060 metabolites were identified across the four quinoa seedling vegetable varieties, encompassing 242 amino acids and derivatives, 277 phenolic acids, 85 nucleotides and derivatives, 497 flavonoids, 23 quinones, 104 lignans and coumarins, 9 tannins, 299 alkaloids, 373 terpenoids, 84 organic acids, 23 steroids, 269 lipids, and 321 unclassified metabolites. Detailed information regarding all identified metabolites is available in Supplementary Table S1. Among these metabolites, flavonoids represented the highest proportion (19.07%), followed by terpenoids (14.31%), alkaloids (11.47%), phenolic acids (10.63%), and lipids (10.32%) (Fig. 4A).

Principal component analysis (PCA) showed that the first principal component (PC1) and the second principal component (PC2) explained 43.12% and 20.91% of the total variance, respectively (Fig. 4B). The four cultivars were clearly separated on the score plot, with the R group exhibiting the most distinct separation from the other three groups along PC1, indicating a unique metabolic profile for the R group. Hierarchical clustering heatmap further confirmed this trend, as samples from the R group clustered together and were distinctly separated from the other groups (Fig. 4C). Therefore, the R group was used as the reference group for subsequent differential metabolite analysis.

### 3.5 Screening of differential metabolites

**Fig. 5:**
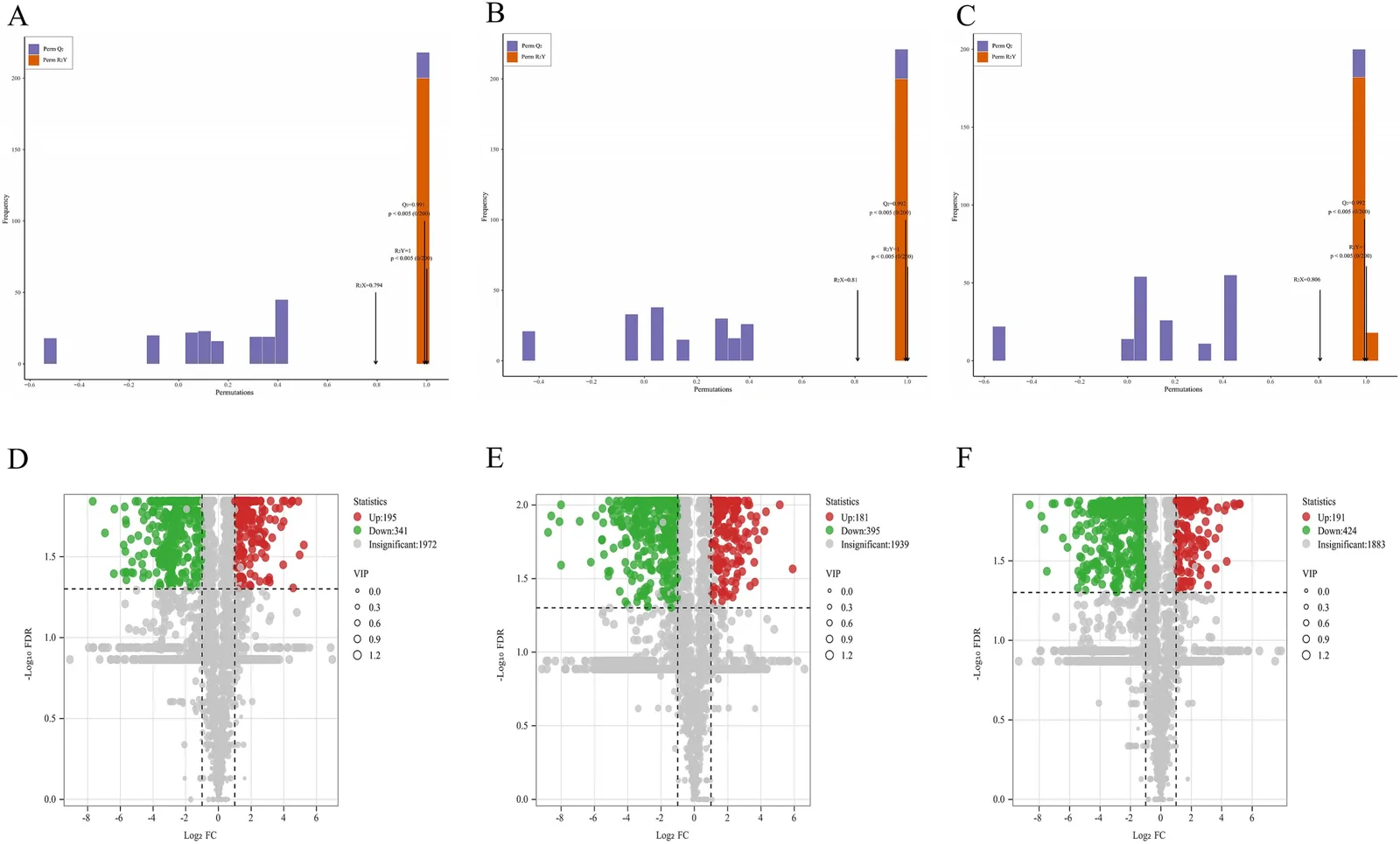
(A) Validation of the orthogonal partial least squares-discriminant analysis (OPLS-DA) model for R vs B; (B) Validation of the OPLS-DA model for R vs Y; (C) Validation of the OPLS-DA model for R vs W. (D) Volcano plot of differential metabolite screening results for R vs B; (E) Volcano plot of differential metabolite screening results for R vs Y; (F) Volcano plot of differential metabolite screening results for R vs W. Dot colors denote differential trends: red for significantly upregulated metabolites, green for significantly downregulated metabolites, and gray for metabolites with no significant differences. (For interpretation of the references to colour in this figure legend, the reader is referred to the web version of this article.)

Orthogonal partial least squares-discriminant analysis (OPLS-DA) is a supervised multivariate statistical pattern recognition method that separates group-related variation from irrelevant background signals and helps visualize intergroup metabolic separation Using Group R as the core group, pairwise OPLS-DA analyses were performed across the four groups to generate score plots, evaluating the metabolic differences between Group R and Group B (R^2^X = 0.794, R^2^Y = 1, Q^2^Y = 0.991; Fig. 5A), Group R and Group Y (R^2^X = 0.81, R^2^Y = 1, Q^2^Y = 0.992; Fig. 5B), and Group R and Group W (R^2^X = 0.806, R^2^Y = 1, Q^2^Y = 0.992; Fig. 5C). We performed 200 permutation tests for each pairwise model, and all comparison pairs exhibited Q^2^ values above 0.9 with permutation test *p*-values less than 0.005. These indicators only reflect the overall grouping trends of the sample.

**Fig. 6:**
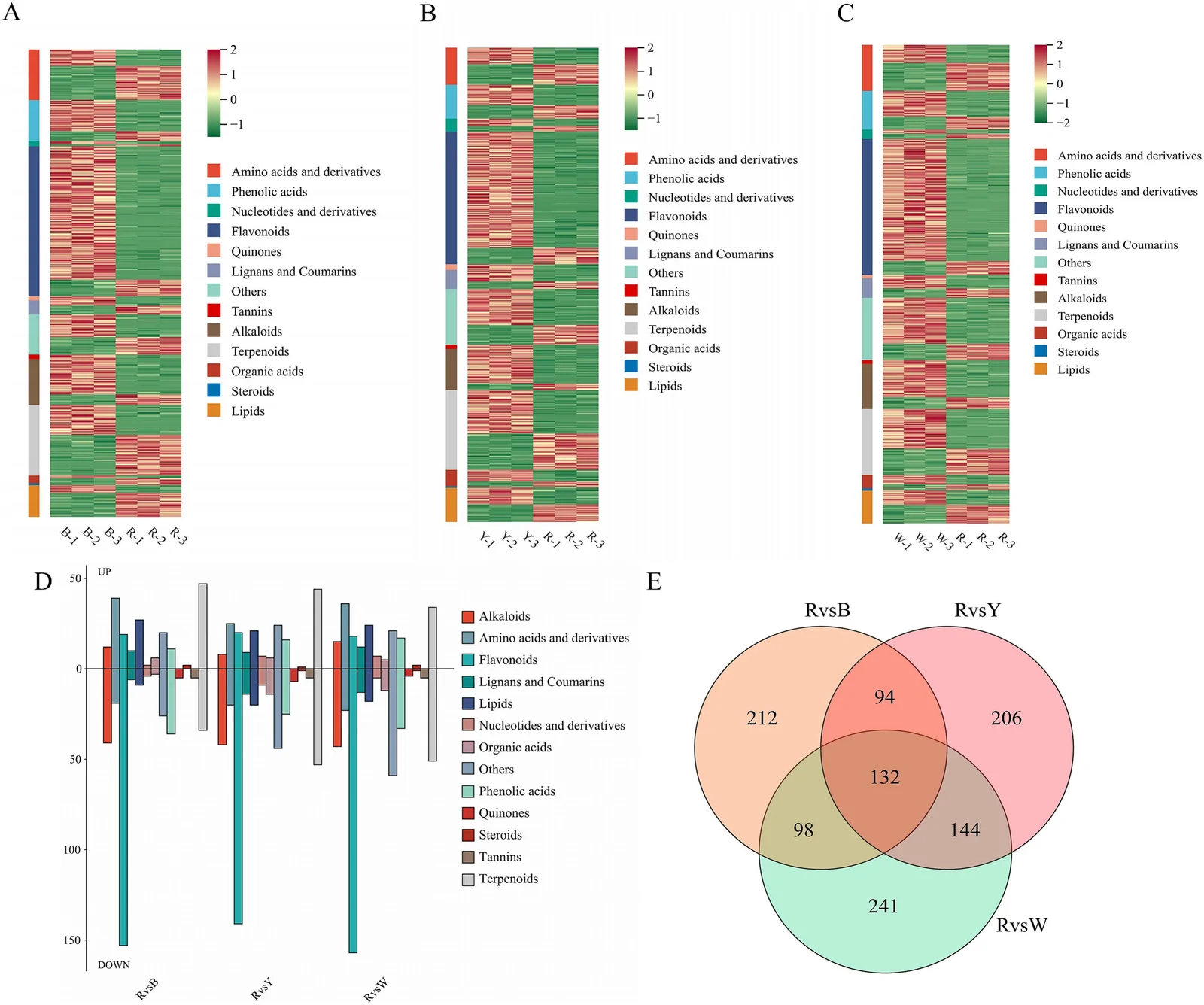
(A) Heatmap of differential metabolite abundances for the R vs B comparison; (B) Heatmap of differential metabolite abundances for the R vs Y comparison; (C) Heatmap of differential metabolite abundances for the R vs W comparison; (D) Mirrored bar chart showing the counts of upregulated and downregulated differential metabolites across different categories in the three comparison pairs; (E) Venn diagram illustrating the intersection analysis of differential metabolites across the three comparison pairs (R vs W, R vs Y, R vs B).

**Fig. 7:**
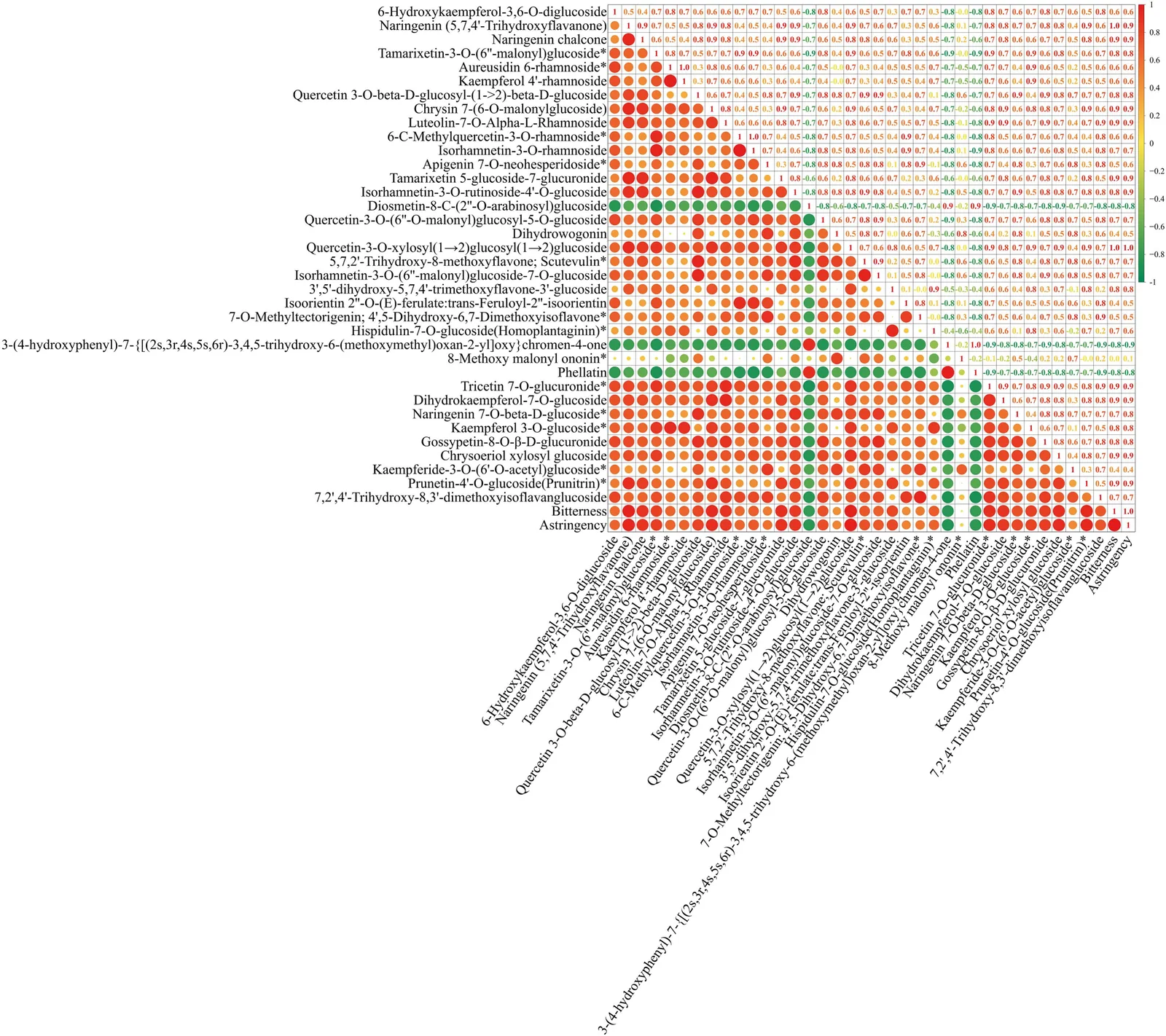
Correlation visualization plot between shared flavonoid differential metabolites (across the three comparison pairs) and the intensities of bitterness and astringency.

Differential metabolites were screened mainly relying on fold change (FC) and FDR-corrected *P*-values, while variable importance in projection (VIP) from OPLS-DA served as an auxiliary screening parameter. A metabolite was defined as differential if it simultaneously satisfied the criteria: FC ≥ 2 or ≤ 0.5, VIP ≥ 1, and FDR-corrected *p*-value <0.05. Detailed information on differential metabolites is provided in Supplementary Table S2. The results showed that 536 significantly differential metabolites were identified between Group R and Group B (195 upregulated, 341 downregulated; Fig. 5D), 576 between Group R and Group Y (181 upregulated, 395 downregulated; Fig. 5E), and 615 between Group R and Group W (191 upregulated, 424 downregulated, Fig. 5F). Furthermore, the number of downregulated metabolites exceeded that of upregulated metabolites across all three comparison pairs. Clustering heatmaps of pairwise comparisons of differential metabolites are presented in Fig. 6A–C. The results revealed that differential metabolites were predominantly enriched in Flavonoids, Alkaloids, and Terpenoids. Fig. 6D displays a mirrored bar chart of differential metabolites across the three comparison pairs. The results demonstrated that, for Flavonoids, Alkaloids, and Phenolic acids, the number of downregulated metabolites exceeded that of upregulated ones in all three comparison pairs; notably, the number of downregulated metabolites in Flavonoids was significantly higher than in the other categories. The Venn diagram (Fig. 6E) illustrates that 241 differential metabolites were unique to the R vs W comparison, 206 to the R vs Y comparison, and 212 to the R vs B comparison. Among pairwise comparisons, 98 differential metabolites were shared between R vs W and R vs B, 144 between R vs W and R vs Y, and 94 between R vs Y and R vs B. Additionally, 132 core differential metabolites were common to all three comparison pairs. As indicated in Supplementary Table S3, Flavonoids accounted for the highest proportion among the shared differential metabolites. Collectively, these findings suggest that Flavonoids may serve as key bitter-tasting compounds in quinoa sprouts. To further elucidate the association between individual flavonoid components and bitter/astringent tastes, a correlation analysis was performed (Fig. 7). The results revealed that most flavonoid glycosides exhibited a significant positive correlation with bitterness and astringency. Among these, the three metabolites with the strongest correlations were Quercetin-3-O-xylosyl(1 → 2)glucosyl(1 → 2)glucoside, Naringenin, and Prunetin-4’-O-glucoside—all of which showed correlation coefficients >0.9 with bitter and astringent tastes. These findings suggest that these metabolites may act as key taste-active compounds contributing to the bitter and astringent flavor of the samples, playing a dominant role in shaping sensory quality. Additionally, metabolites strongly correlated with bitter and astringent tastes were predominantly flavonol compounds, suggesting that flavonols may be the primary category of metabolites contributing to bitter and astringent flavors. Notably, a perfect positive correlation (correlation coefficient = 1.00) was observed between bitterness and astringency in the figure, indicating that these two sensory attributes varied in synchrony across the samples and exhibited a high degree of synergy. This fully coordinated trend is speculated to be largely associated with the inherent properties of the quinoa seedling matrix.

### 3.6 Metabolic pathway analysis of differential metabolites

**Fig. 8:**
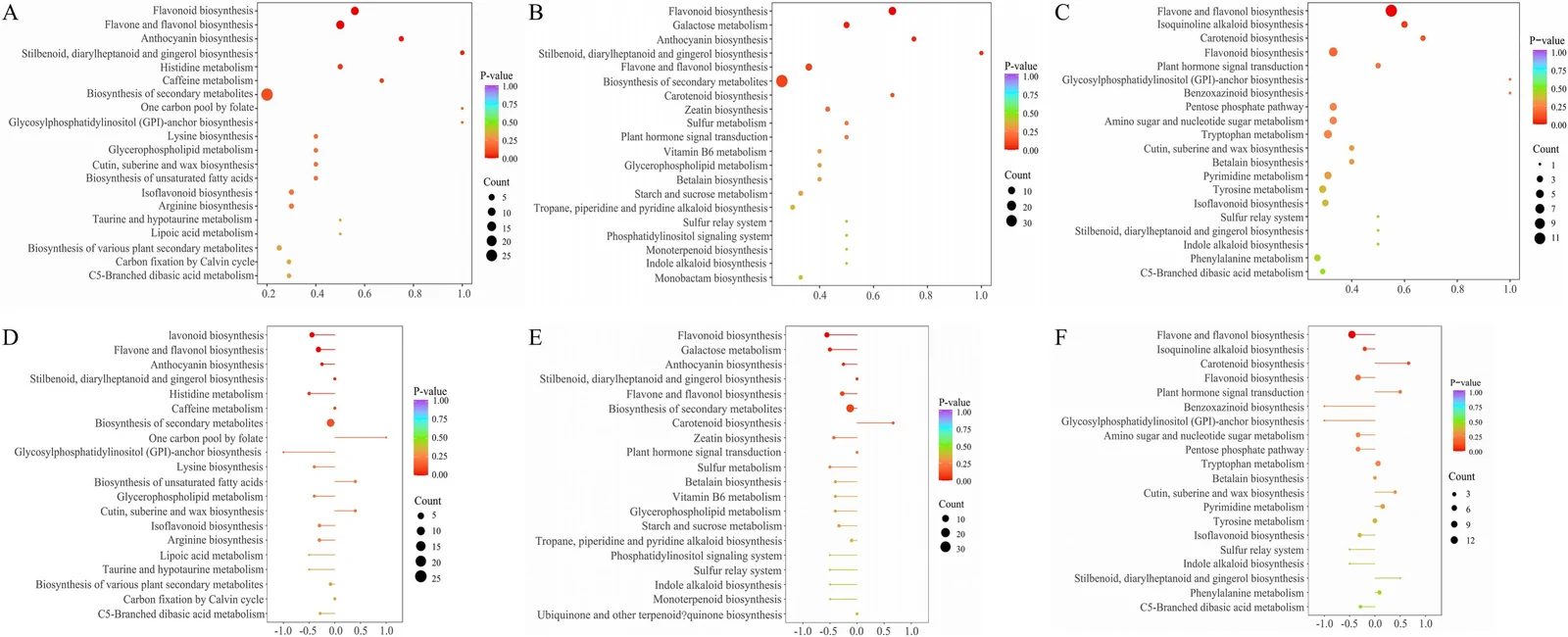
(A) Pathway enrichment results for R vs B; (B) Pathway enrichment results for R vs Y; (C) Pathway enrichment results for R vs W; (D) Differential abundance analysis results of enriched metabolic pathways for R vs B; (E) Differential abundance analysis results of enriched metabolic pathways for R vs Y; (F) Differential abundance analysis results of enriched metabolic pathways for R vs W.

All differential metabolites across the comparison pairs were mapped to the KEGG database to retrieve pathway annotations. Enrichment analysis was then performed on the annotated results to identify pathways significantly enriched with differential metabolites. For the R vs B comparison (Fig. 8A), the enrichment results indicated that Flavonoid biosynthesis and Flavone and flavonol biosynthesis were the most significantly enriched pathways, followed by Biosynthesis of secondary metabolites, Anthocyanin biosynthesis, and Stilbenoid, diarylheptanoid and gingerol biosynthesis. For the R vs Y comparison (Fig. 8B), Flavonoid biosynthesis was the most significantly enriched pathway, followed by Galactose metabolism, Anthocyanin biosynthesis, Stilbenoid, diarylheptanoid and gingerol biosynthesis, and Flavone and flavonol biosynthesis. For the R vs W comparison (Fig. 8C), Flavone and flavonol biosynthesis ranked as the most significantly enriched pathway, followed by Isoquinoline alkaloid biosynthesis, Carotenoid biosynthesis (duplicated entry noted), Flavonoid biosynthesis, and Plant hormone signal transduction. Cross-comparison of metabolic pathway enrichment results across the three comparison pairs revealed that Flavonoid biosynthesis, Flavone and flavonol biosynthesis, and Stilbenoid, diarylheptanoid and gingerol biosynthesis were significantly enriched in all three comparisons. Notably, Flavonoid biosynthesis and Flavone and flavonol biosynthesis showed statistically significant differences across all three comparison pairs. To evaluate the global regulatory trends of metabolic pathways across the three comparison pairs, pathway-level differential analysis was performed using the Differential Abundance Score (DA Score) (Fig. 8D–F). Results indicated that the Flavonoid biosynthesis pathway and the Flavone and flavonol biosynthesis pathways exhibited consistent overall downregulation across all three comparison pairs. In contrast, the Stilbenoid, diarylheptanoid, and gingerol biosynthesis pathways displayed divergent regulatory trends across the three comparisons. These findings suggest that key bitter and astringent compounds in quinoa sprouts likely derive from the Flavonoid biosynthesis and the Flavone and flavonol biosynthesis pathways.

**Fig. 9:**
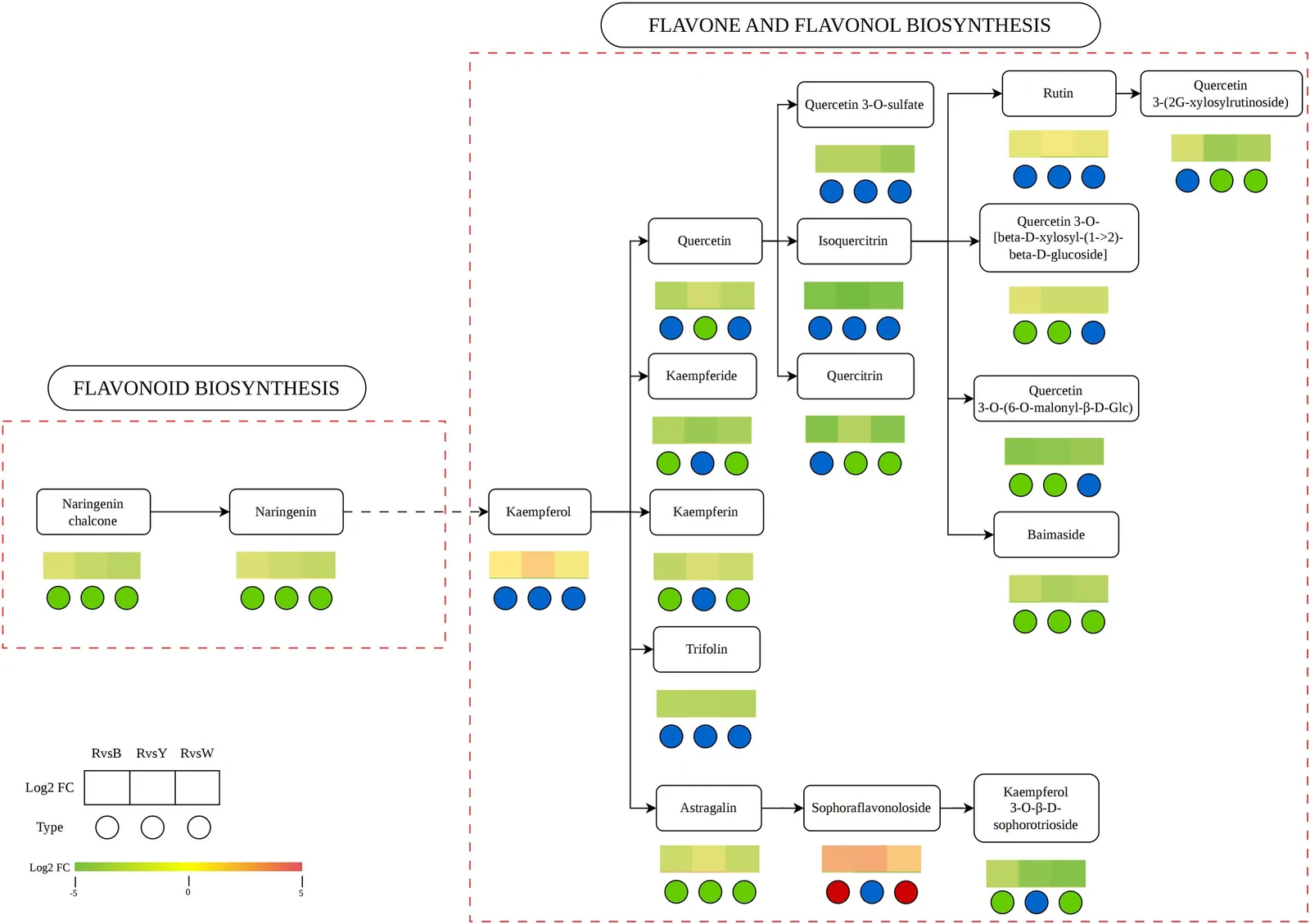
Enrichment Pathway Map of Core Differential Metabolites Among Three Comparison Groups. Red indicates significant upregulation in the comparison pair, green indicates significant downregulation, and blue indicates no significant difference. (For interpretation of the references to colour in this figure legend, the reader is referred to the web version of this article.)

Key flavonoid derivatives linked to bitter–astringent flavor are compiled in Supplementary Table S4, and their correlation with the bitter–astringent sensation of quinoa seedlings is further verified by the enrichment pathway map of core differential metabolites (Fig. 9). Results indicated that naringenin chalcone and naringenin, components of the Flavonoid biosynthesis pathway, showed consistent downregulation across all three comparison pairs. Additionally, these two metabolites are localized upstream of the Flavone and flavonol biosynthesis pathway, and their relative abundance trends were consistent with the trend of bitter and astringent taste intensity. In contrast, differential changes in the Flavone and flavanol biosynthesis pathways were concentrated in two branch pathways: quercetin aglycone biosynthesis and kaempferol aglycone biosynthesis. Notably, astragalin and baimaside exhibited a consistent downregulation trend across the three comparison pairs in the respective pathways. These findings suggest that the key bitter and astringent compounds in quinoa sprouts are likely associated with quercetin aglycone and kaempferol aglycone, and the bitter and astringent taste of quinoa seedling vegetables is synergistically induced by quercetin and kaempferol.

## 4 Discussion

Quinoa seedlings are an emerging leafy vegetable with high nutritional value (Gómez et al., 2024). However, their intrinsic bitterness and astringency may limit consumer acceptance (Li et al., 2025). Bitterness primarily arises from secondary metabolites such as alkaloids, terpenoids, amino acid derivatives, fatty acid derivatives, and flavonoids (Liu et al., 2024; Zhou et al., 2024). In contrast, astringency is mainly attributed to polyphenols—including flavonoids, phenolic acids, and tannins—and may also stem from metal ion salts, acids (both organic and inorganic), and other substances (Liu et al., 2023). Research has indicated that the bitter and astringent taste of quinoa grains is primarily attributed to saponins (Guo et al., 2024; Suárez-Estrella et al., 2021). In contrast, emerging studies suggest that the bitterness of quinoa grains mainly stems from flavonoids and polyphenolic compounds (Song et al., 2024). From this, it can be inferred that the bitter and astringent taste exhibited by quinoa seedling vegetables is most likely due to saponins, flavonoids, and polyphenolic compounds. Based on the findings of this study, total polyphenols, flavonoids, alkaloids, and terpenoids all account for a substantial proportion of quinoa seedling vegetables, suggesting their potential association with bitterness and astringency. However, the intensity trends of bitterness and astringency across the four groups of quinoa seedling vegetable samples align with the content trends of total polyphenols and flavonoids, but not with those of alkaloids and saponins. Alkaloids and saponins may contribute relatively little to the bitterness and astringency of quinoa seedling vegetables. Furthermore, the bitterness is primarily attributed to physcion derivatives within saponins (Guo et al., 2024). While no significant difference in saponin content was observed between quinoa seedlings and seeds, the saponin aglycone content in seedlings is substantially lower than that in seeds—particularly the content of physcion derivatives (Lim et al., 2020). This further suggests that the bitterness and astringency in quinoa seedling vegetables are unlikely to be primarily due to saponins. Thus, it is highly probable that the bitterness and astringency in quinoa seedling vegetables also originate from flavonoids and polyphenolic compounds.

According to data from the widely targeted metabolomics approach, flavonoid metabolites accounted for a relatively high proportion and represented the largest share among common metabolites across the three comparison groups. Additionally, flavonoids are a subclass of polyphenols (Beecher, 2003), which suggests that the bitterness and astringency of quinoa seedling vegetables may primarily originate from flavonoids. It has been reported that flavonoids make a significant contribution to the bitterness of fruits, vegetables, and beverages (Yan & Tong, 2023). Furthermore, monomeric flavonoids are predominantly bitter, whereas polymeric flavonoids are primarily astringent; notably, the rate of increase in astringency exceeds that of bitterness as the degree of polymerization rises (Given & Paredes, 2002). Notably, bitter and astringent flavors exhibit a synergistic relationship, as most bitter substances are also classified as astringent at the level of taste perception (Chen et al., 2022; Deng et al., 2022). Flavonoids can bind to oral bitter taste receptors (the *hTAS2R* family) to elicit bitterness (Roland et al., 2013). Flavonoids form complexes with proline-rich proteins (PRPs) in saliva, leading to precipitation and the generation of astringency (Given & Paredes, 2002). The structure of flavonoid glycosides—including the aglycone core, sugar type, and sugar sequence—is also associated with astringency intensity (Scharbert et al., 2004). These findings indicate that flavonoids are capable of inducing both bitterness and astringency. Furthermore, based on the results of the differential metabolite enrichment analysis, flavonoid compounds showed a significant correlation with the bitter and astringent sensory indicators of quinoa seedling vegetables. It is speculated that these compounds may be the potential key metabolites closely related to the perception of bitterness and astringency. In the present study, naringenin showed a strong correlation with bitterness and astringency; however, previous research has confirmed that naringenin itself does not possess a bitter taste (Huang et al., 2025). Notably, naringenin serves as an upstream precursor in the biosynthetic pathways of flavones and flavonols (Cao et al., 2024). Specifically, naringenin, kaempferol, and quercetin constitute a linear metabolic cascade characterized by the sequence “precursor → intermediate → downstream product” (Tartik et al., 2023). Based on the above correlational results, we put forward two speculative hypotheses for hesperidin: it may interact with taste receptors to trigger bitter and astringent perception, or it may represent an intermediate metabolite that indirectly reflects the overall accumulation of downstream flavor-active compounds associated with bitter and astringent taste within the metabolic pathway. The flavone and flavonol biosynthetic pathways, enriched in differential metabolites, represent core branches of the broader flavonoid biosynthetic pathway (W. Liu et al., 2021). Furthermore, existing studies have shown that the flavone and flavanol biosynthetic pathways are strongly associated with bitterness and astringency (Jiang et al., 2023). Notably, both flavones and flavonols—two critical subfamilies of flavonoids—are also closely linked to bitterness and astringency (Jin et al., 2024; Liu et al., 2023). Integrating the analysis results for shared differential metabolites across the three comparison groups in the present study and the highly significantly enriched pathways of differential metabolites, it can be concluded that bitterness and astringency are closely associated with flavanol metabolites. In the present study, most variations in the flavone and flavonol biosynthetic pathways were concentrated in two branch pathways: quercetin aglycone biosynthesis and kaempferol aglycone biosynthesis. All metabolites involved in these two branches belong to the flavonol class. The above correlation results imply that the flavonols quercetin and kaempferol are closely associated with bitter and astringent taste traits and may serve as key metabolites involved in the formation of bitter and astringent sensations. Quercetin and kaempferol are both capable of activating the human bitter taste receptors *hTAS2R14* and *hTAS2R39* (Karolkowski et al., 2023). Additionally, several glycoside derivatives of quercetin and kaempferol have been identified as key contributors to bitterness and astringency (Deng et al., 2022; Jin et al., 2024). In the present study, no significant differences were observed in the contents of quercetin and kaempferol across the four quinoa seedling vegetable samples, whereas their glycoside derivatives exhibited significant variations in content. Notably, astragalin—an important metabolite identified in this study—has been validated as a bitterness and astringency-active component, with a more pronounced contribution to astringency (Walser et al., 2024). Although baimaside has not been explicitly linked to bitterness or astringency, as a glycoside derivative of quercetin, it holds the potential to induce bitterness and astringency in quinoa seedling vegetables. These findings suggest that these metabolites may not exert their taste-regulating effects independently; instead, they might jointly shape the sample's bitter and astringent sensory characteristics through coordinated regulation.

In conclusion, it is speculated that flavonoids are the potential key metabolites related to the perception of bitterness and astringency in quinoa seedling vegetables. In parallel, the coexistence of polysaccharides, proteins, and saponins in the matrix may enhance the solubility of flavonoid compounds, thereby intensifying the inherent taste response (Liu et al., 2020). Notably, although these flavonol glycosides are associated with undesirable taste attributes, they are well documented for their antioxidant, anti-inflammatory, and cardioprotective effects (Jan et al., 2022).From an application perspective, based on the correlation analysis results of this study, merely reducing the content of saponins may not significantly improve the palatability of quinoa seedling vegetables. Since there is a significant statistical correlation between flavonols and the bitter sensory characteristics, regulating the biosynthesis or glycosylation pathways of flavonols can be initially considered as a potential research direction for flavor improvement breeding. However, these flavonoid glycosides can only be considered as potential candidate metabolites involved in the formation of bitterness and astringency, rather than functionally validated compounds responsible for bitter or astringent activity. Further, it is necessary to combine sensory recombination verification, taste receptor level detection and other experiments to systematically clarify the real flavor contribution mechanisms of various flavonol glycosides.

## 5 Conclusion

This study combined electronic tongue detection and widely targeted metabolomics to explore the chemical factors linked to bitter and astringent taste across four quinoa seedling cultivars. Correlation analysis revealed that flavonoids, especially quercetin and kaempferol glycosides, showed strong statistical associations with bitter and astringent sensory signals, and are speculated to be potential candidate metabolites related to these unpleasant taste properties. The above correlation results have enriched our understanding of the potential chemical factors related to the bitter and astringent flavors of quinoa seedling vegetables. The significant statistical association between flavonoids and the undesirable flavor characteristics provides preliminary clues for the possible correlation with sensory properties, and at the same time offers a basic reference for breeding varieties of quinoa seedling vegetables with low bitterness and astringency.

## CRediT authorship contribution statement

**Jie Yang:** Writing – original draft, Investigation, Data curation. **Yuanyuan Zuo:** Writing – original draft, Formal analysis. **Chen Chen:** Validation. **Hanxue Li:** Conceptualization. **Xuqin Wang:** Methodology. **Guofei Jiang:** Software. **Yuming Xie:** Investigation. **Huanyao Xia:** Investigation. **Jianlong Zhou:** Validation. **Lurongrong Gao:** Visualization. **Yutao Bai:** Resources. **Chuanli Zhang:** Writing – review & editing, Project administration. **Peng Qin:** Writing – review & editing, Funding acquisition.

## Ethics declaration

The author(s) declare(s) that the study does not involve humans nor animal subjects.

## Declaration of competing interest

The authors declare that they have no known competing financial interests or personal relationships that could have appeared to influence the work reported in this paper.

## Data Availability

Data will be made available on request.

## Appendix A Supplementary data

Supplementary material 1 Metabolite ID, annotated name, and peak area of metabolites detected in four quinoa seedling vegetables under different treatments (Supplementary Table S1).

Supplementary material 2 Detailed information of differential metabolites identified in four quinoa seedling vegetables (Supplementary Table S2).

Supplementary material 3 Information of common differential metabolites across three comparison groups in four quinoa seedling vegetables (Supplementary Table S3).

Supplementary material 4 Key flavonoid derivatives linked to bitter astringent flavor in four quinoa seedling vegetables (Supplementary Table S4).
